# Polymorphism of the insulin gene is associated with increased prostate cancer risk

**DOI:** 10.1038/sj.bjc.6600747

**Published:** 2003-01-28

**Authors:** G Y F Ho, A Melman, S-M Liu, M Li, H Yu, A Negassa, R D Burk, A W Hsing, R Ghavamian, S C Chua

**Affiliations:** 1 Department of Epidemiology and Social Medicine, Albert Einstein College of Medicine, Bronx, New York, NY 10461, USA; 2 Department of Urology, Albert Einstein College of Medicine, Bronx, New York, NY 10461, USA; 3 Department of Pathology, Albert Einstein College of Medicine, Bronx, New York, NY 10461, USA; 4 Department of Pediatrics, Albert Einstein College of Medicine, Bronx, New York, NY 10461, USA; 5 Division of Molecular Genetics, Department of Pediatrics, Columbia University, New York, NY 10032, USA; 6 Department of Epidemiology and Public Health, Yale University, New Haven, CT 06520, USA; 7 Division of Cancer Epidemiology and Genetics, National Cancer Institute, Bethesda, MD 20852, USA

**Keywords:** prostate cancer, insulin, polymorphism

## Abstract

High insulin levels are linked with increased cancer risk, including prostate cancer. We examined the associations between prostate cancer with polymorphisms of the insulin gene (*INS*) and its neighbouring genes, tyrosine-hydroxylase and IGF-II (*TH* and *IGF2*). In this study, 126 case–control pairs matched on age, race, and countries of origin were genotyped for +1127 *INS-Pst*I in *INS*, –4217 *TH-Pst*I in *TH*, and +3580 *IGF2-Msp*I in *IGF2*. The homozygous CC genotype of +1127 *INS-Pst*I occurred in over 60% of the population. It was associated with an increased risk of prostate cancer in nondiabetic Blacks and Caucasians (OR=3.14, *P*=0.008). The CC genotype was also associated with a low Gleason score <7 (OR=2.60, *P*=0.022) and a late age of diagnosis (OR=2.10, *P*=0.046). Markers in the neighbouring genes of *INS* showed only null to modest associations with prostate cancer. The polymorphism of *INS* may play a role in the aetiology of prostate cancer. Given the high prevalence of the CC genotype and its association with late age of onset of low-grade tumours, this polymorphism may contribute to the unique characteristics of prostate cancer, namely a high prevalence of indolent cancers and the dramatic increase in incidence with age.

The insulin-like growth factor (IGF) system, which includes two ligands (IGF-I and IGF-II), two cell membrane receptors (IGF-1R and IGF-2R), six binding proteins (IGFBP-1 through IGFBP-6), and a large group of IGFBP proteases (Grimberg and Cohen, 2000; Khandwala *et al*, 2000; Yu and Rohan, 2000), has been implicated in carcinogenesis because of its important role in regulating cell proliferation, differentiation, apoptosis, and transformation (Grimberg and Cohen, 2000). There are several consistent reports that link risk of prostate cancer with high serum levels of IGF-I (Mantzoros *et al*, 1997; Chan *et al*, 1998; Wolk *et al*, 1998; Stattin *et al*, 2000; Chokkalingam *et al*, 2001). Decreased levels of IGFBP-3, the most abundant IGFBP in the circulation, have been found in prostate cancer patients and in those with metastatic diseases (Kanety *et al*, 1993; Chan *et al*, 1998; Chokkalingam *et al*, 2001).

Insulin is hypothesised as a risk factor of prostate cancer because of its structural and regulatory relations with the IGF system. There is structural homology among insulin, IGF-I, and IGF-II as well as between insulin receptor and IGF-1R (Khandwala *et al*, 2000; Yu and Rohan, 2000), so insulin and IGFs can crossbind to each other's receptor although with weak affinity (Efstratiadis, 1998). Insulin regulates IGFBP-1 and may affect circulating levels of free IGFs (Powell *et al*, 1991). In addition to its close relation with the IGF system, the negative correlation between high insulin and decreased sex hormone-binding protein (SHBP) may result in increased levels of free testosterone (Strain *et al*, 1994; Pasquali *et al*, 1995). So far, only one study in prostate cancer has examined serum insulin levels using fasting blood. In this case–control study conducted in China, men with insulin levels in the highest tertile had a 2.5-fold increased risk of prostate cancer compared to men in the lowest tertile after adjusting for IGF-I and anthropometric factors (Hsing *et al*, 2001). One of the causes for elevated insulin levels might be genetic variation in the insulin gene (*INS*). We examined the relation between risk of prostate cancer and a single nucleotide polymorphism (SNP) marker in *INS* in a case–control study.

The insulin gene is located on chromosome 11 (11p15.5). The variable number of tandem repeat (VNTR) that lies immediately adjacent to the 5′ promoter region of *INS* is believed to have a direct effect on *INS* regulation (Kennedy *et al*, 1995). The polymorphism of the VNTR can be classified into two main groups: small class I alleles (28–44 repeats) and large class III alleles (138–159 repeats) at frequencies of about 70 and 30%, respectively, and class II alleles of intermediate size are rare (Stead and Jeffreys, 2000). The class I allele is associated with increased expression of insulin mRNA and insulin levels (Lucassen *et al*, 1995; Bennett and Todd, 1996; Le Stunff *et al*, 2000). The allelic variation of VNTR is also associated with the risk of diabetes. It has been found consistently that the class I allele increases the risk of type I diabetes (Julier *et al*, 1991; Lucassen *et al*, 1993; Bennett and Todd, 1996). An association between the class III allele and type II diabetes has also been reported (Ong *et al*, 1999). In addition to the VNTR, there are 10 noncoding SNP markers that span the 4.1 kb segment of the entire *INS* gene and its flanking intergenic regions. It has been shown in Caucasian populations that these 10 SNPs are in tight linkage disequilibrium with each other and with the VNTR such that they constitute two major haplotypes (Cox *et al*, 1988; Julier *et al*, 1991; Lucassen *et al*, 1993). In such a region of tight linkage disequilibrium, assaying for one marker would generally provide genotype information of all the others. We assayed for two of these SNP markers, the +1127 *INS-Pst*I and +1428 *INS-Fok*I (the positive number indicates the number of base pairs downstream from the initiator codon of *INS*), as the surrogates for the VNTR. We found complete concordance for genotypes at these two markers in 50 subjects tested. Here, we reported the results of the *Pst*I marker in the entire study population. The *Pst*I marker was chosen for genotyping, since it was reported to be in complete linkage disequilibrium with three other SNPs in the *INS* genomic region, and genotypes for this marker were almost always identical to those at the VNTR (Lucassen *et al*, 1993). To further show that *INS*, rather than adjacent genes on chromosome 11, is indeed the risk-associated gene, we also genotyped two markers in the tyrosine-hydroxylase and IGF-II genes (*TH* and *IGF2*), which flank the 5′ and 3′ ends of *INS*, respectively (Figure 1

**Figure 1:**
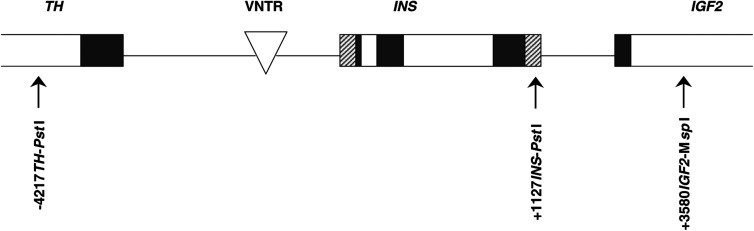
Position of SNPs examined in the *TH-INS-IGF2* region on chromosome 11p15.5. This 19-kb genomic region, in which 19 polymorphisms have been identified, includes the tyrosine hydroxylase (*TH*), insulin (*INS*), and IGF-II (*IGF2*) genes (Lucassen *et al*, 1993). Three SNPs were examined in this study and are designated by their position (the positive and negative numbers indicate the number of base pairs downstream and upstream from the initiator codon of *INS*, respectively), corresponding gene, and the restriction enzyme used for detection: (a) –4217 T/C polymorphism of *TH* detected by *Pst*I, (b) +1127 T/C polymorphism of *INS* detected by *Pst*I, and (c) +3580 G/A polymorphism of *IGF2* detected by *Msp*I. Open, closed, and hatched boxes refer to introns, exons, and untranslated regions, respectively. The triangle represents the variable number of tandem repeat (VNTR) locus adjacent to the promoter region of *INS*. Figure not drawn to scale.

).

## MATERIALS AND METHODS

In this case–control study, cases with histopathologically confirmed prostate cancer were identified from two hospitals affiliated with the Albert Einstein College of Medicine. They were private patients either diagnosed or treated at the Departments of Urology in these hospitals. After obtaining a physician's approval, sequential patients who had no history of other cancers were recruited within a year of diagnosis. For potential controls, outpatients who were male and ⩾40 years old were randomly sampled from the billing records of the Departments of Medicine in the same hospitals where the cases arose. Patients who had no history of any cancer and had intact prostate and testes formed a pool of eligible controls. The reasons for seeing an internist were not used as exclusion criteria. One control was matched to each case on birth year (±5 years), race, and countries of origin. Questionnaire data and nonfasting blood samples were obtained. DNA was extracted from whole blood and genotyped for three markers in the *TH-INS-IGF2* region (Figure 1) by a polymerase chain reaction-based restriction fragment length polymorphism assay (PCR-RFLP). The study protocol was approved by the institutional review board, and informed consent was obtained from all subjects.

Between 1998 and 2000, 191 cases and 148 controls were recruited. Genotyping was performed on 178 (93%) cases and 135 (91%) controls who had a DNA sample. Odds ratios for the associations between polymorphisms and risk of prostate cancer were estimated by conditional logistic regression in 126 case–control pairs. A total of 52 cases were excluded from conditional logistic regression analyses either because a control who fulfilled the three matching factors was not available or the matched control did not have genotype data. They were not different from the 126 analysable cases in terms of age at diagnosis, Gleason score, race, and genotype frequencies. Moreover, using all the recruited cases and controls in unmatched analyses yielded similar results. The results from conditional logistic regression were presented, since it is the appropriate and standard statistical method for analysing matched case–control data.

To examine the effects of the *INS* polymorphism on two diagnostic characteristics, namely Gleason score (<7 or ⩾7) and age at diagnosis (<55, 55–64, or ⩾65), case–case analyses were performed among all the 178 cases. The cut point for Gleason score was chosen for its association with tumour extent, prognosis, and survival (Kattan *et al*, 1997; D'Amico *et al*, 1998). Treating Gleason score and age at diagnosis as the outcome variables, logistic regression analyses for dichotomous and ordinal dependent variables, respectively, were performed to examine their associations with the *INS* polymorphism while controlling for confounding variables. Data were analysed by the statistical software package SAS (SAS Institute Inc., 1989). All *P*-values presented are two-sided.

Three SNPs in three genes were genotyped in this study. To measure the extent of linkage disequilibrium between any pairwise markers, we first inferred phase and reconstructed haplotypes using the PHASE software (http://www.stat.washington. edu/stephens/phase.html) developed by Stephens *et al*,(2001). Linkage disequilibrium was measured by *D*′ (Devlin and Risch, 1995).

### PCR-RFLP assays for detection of polymorphisms

Primers to amplify the three genomic regions are:

*INS*+1035: GGG TCC CCT GCA GAA GCG TGG CA
*INS*+1597: CTC CCT CCA CAG GGA CTC CAT C
*TH-Pst*F: TGA CGC CAA GGA CAA GCT CAG GT
*TH-Pst*R: CCA CCC AGC AGC CCC AGT CCT GT
*IGF2-Msp*F: CCA CCC CTT CTG GGA AGC TAA AAG
*IGF2-Msp*R: CCC TCG GTC CTC CAG GAA TGG ACA

The *TH* and *IGF2* amplicons were amplified using standard Taq DNA polymerase with cycling plateaus of 94°–55°–72° for 30 seconds each (35 cycles). The *INS* amplicon was more refractory to reliable amplification with Taq DNA polymerase. We used LA Taq, a DNA polymerase mix, with a proofreading enzyme in order to obtain reliable amplification. Amplified DNA (5 *μ*l) was used in a 10 *μ*l restriction enzyme digest with 1–2 U of enzyme using the manufacturer's recommendations. Digested products were size fractionated on high percentage agarose gels and visualised by UV-induced ethidium fluorescence. The restriction fragments of the alleles are as follows:

*INS*: T allele 562 bp
C allele 470 bp+92 bp
*TH*: T allele 240 bp
C allele 148 bp+92 bp
*IGF2*: A allele 122 bp+118 bp
G allele 122 bp+84 bp+34 bp

## RESULTS

The age at diagnosis of the prostate cancer cases ranged from 43 to 88 years, with a median at 63. The majority of the cases (77%) were diagnosed due to an abnormal PSA test or digital rectal examination. The ethnic distribution of the cases was 54% African Americans, 22% Caucasians, 21% Hispanics, and 3% others, reflecting the ethnic distribution of the population in the catchment area. Genotype frequencies of the three markers in the *TH-INS-IGF2* region by ethnicity are presented in Table 1

**Table 1:** Percent distributions of the genotypes and pairwise linkage disequilibrium scores for three markers in the *TH-INS-IGF2* region stratified by ethnicity

	**All races**^a^		**Black**		**Caucasian**		**Hispanic**	
	**Cases**	**Controls**	**Cases**	**Controls**	**Cases**	**Controls**	**Cases**	**Controls**
	***N*=178 (%)**	***N*=135 (%)**	***N*=96 (%)**	***N*=67 (%)**	***N*=40 (%)**	***N*=36 (%)**	***N*=37 (%)**	***N*=29 (%)**
+1127 *INS-Pst*I								
CC	80	70	81	72	80	61	73	79
CT	18	26	18	27	17	33	24	10
TT	2	4	1	1	3	6	3	10
								
−4217 *TH-Pst*I								
CC	13	17	8	5	25	33	14	25
CT	43	31	39	20	47	42	43	36
TT	44	52	53	75	28	25	43	39
								
+3580 *IGF2-Msp*I								
GG	42	42	50	37	25	49	41	50
AG	40	46	37	53	55	41	32	37
AA	18	11	13	10	20	10	27	13
								
Linkage disequilibrium score (D′)^b^								
+1127 *INS-Pst*I and −4217 *TH-Pst*I			0.76	1	1			
+1127 *INS-Pst*I and +3580 *IGF2-Msp*I			0.85	0.80	0.87			
−4217 *TH-Pst*I and +3580 *IGF2-Msp*I			0.49	0.45	0.31			

a Subjects with mixed ethnicity were included here, but not in the race-specific columns.

b *D*′ was determined with cases and controls combined.

. The +1127 *INS-Pst*I marker was in linkage disequilibrium with both –4217 *TH-Pst*I and +3580 *IGF2-Msp*I in the neighbouring genes (Table 1). The C alleles of –4217 *TH-Pst*I were linked to the C alleles of +1127 *INS-Pst*I, resulting in a linkage disequilibrium score (D′) of one; the two markers, however, were not in complete linkage disequilibrium. For +1127 *INS-Pst*I, the homozygous CC was the predominant genotype. The heterozygous CT and homozygous TT were grouped together as the ‘other genotypes’ in analyses due to small numbers.

Table 2

**Table 2:** Odds ratios for the association between prostate cancer and the homozygous CC genotype of +1127 *INS-Pst*I

	**No of pairs**	**OR (95% CI)**^a^	***P*-value**
All subjects	126	1.74 (0.99–3.05)	0.055
By diabetes status			
No	86	2.18 (1.07–4.45)	0.032
Yes^b^	40	1.13 (0.43–2.92)	0.809
By race, among nondiabetics			
Black	45	2.75 (0.88–8.64)	0.083
Caucasian	26	3.67 (1.02–13.14)	0.046
Hispanic	14	0.25 (0.03–2.24)	0.215
Black and Caucasian, among nondiabetics	71	3.14 (1.34–7.36)	0.008
By age, among nondiabetic Black and Caucasian^c^			
<55	14	0.75 (0.17–3.35)	0.706
⩾55	57	6.33 (1.87–21.4)	0.003

a Odds ratios for the risk of prostate cancer when subjects with homozygous CC were compared with those with other genotypes (the heterozygous and homozygous TT), with the latter as the reference group.

b One or both members of the pair had diabetes.

c The case's age at diagnosis was used for classification of the pair. *P*-value for homogeneity of odds ratios (i.e. interaction between age and the +1127 *INS-Pst*I polymorphism)=0.030.

shows that individuals with homozygous CC for +1127 *INS-Pst*I had almost a two-fold increased risk of prostate cancer as compared to those with other genotypes (OR=1.74). As the polymorphism at +1127 *INS-Pst*I and its tightly linked VNTR have been reported to be associated with diabetes (Lucassen *et al*, 1993), we first stratified the analysis by diabetes status, which was based on self-report. There was a disparity by diabetes status, such that the association between +1127 *INS-Pst*I and prostate cancer was apparent among subjects without diabetes (OR=2.18) but not among those with diabetes (OR=1.13). Subsequent analyses were then limited to case–control pairs in which both members were nondiabetic. We then evaluated if there was heterogeneity in disease association by ethnicity. Stratified analyses by ethnicity showed that association existed among the nondiabetic Black subjects (OR=2.75) and Caucasians (OR=3.67), but not the Hispanics (OR=0.25). The sample size for the Hispanics was small, and their genotype frequencies of +1127 *INS-Pst*I were also not in Hardy–Weinberg equilibrium. The genetic effect was age-dependent: the strongest association between the CC genotype and increased risk of prostate cancer occurred among subjects who were Black or Caucasian and ⩾55 years old (ORs for <55, 55–64, and ⩾65=0.75, 5.0, and 9.0, respectively).

Table 1 showed that linkage disequilibrium existed between +1127 *INS-Pst*I and the two markers in the flanking neighbouring genes. It is possible that a locus adjacent to *INS* is in fact the disease-associated gene, and the observed association with +1127 *INS-Pst*I is due to linkage disequilibrium between polymorphisms of the disease-associated gene and *INS*. If so, polymorphism of the disease-associated gene should demonstrate a stronger association with prostate cancer than +1127 *INS-Pst*I. Table 3

**Table 3:** Associations between prostate cancer and three markers in the *TH-INS-IGF2* genomic region – among non-diabetic Black and Caucasian subjects (*n*=68 pairs)

	**Adjusted OR (95% CI)**^a^	***P***
+1127 *INS-Pst*I		
CT or TT	1	
CC	3.57 (1.35–9.45)	0.010
		
−4217 *TH-Pst*I		
TT	1	
CT	2.27 (0.99–5.19)	0.053
CC	1.72 (0.55–5.42)	0.355
		
+3580 *IGF2-Msp*I^b^		
GG	1	
AG	0.90 (0.42–1.95)	0.797
AA	1.52 (0.37–6.27)	0.559

a Odds ratios were adjusted for the other two markers in the model.

b *IGF2* is subject to parental imprinting, and only the paternal allele is expressed. OR for the heterozygous genotype was difficult to interpret, when the expressing allele (A or G) was not identified. Conversely, knowing the active allele among the subjects with homozygous AA or GG was irrelevant. Hence, the OR for the homozygous AA was fully interpretable when the homozygous GG was used as the reference.

shows that prostate cancer remained to have the strongest association with the CC genotype of +1127 *INS-Pst*I (OR=3.57); the associations with –4217 *TH-Pst*I (CT *vs* TT, OR=2.27) and +3580 *IGF2-Msp*I (AA *vs* GG, OR=1.52) were comparatively moderate. Moreover, the association between –4217 *TH-Pst*I and prostate cancer was attributed to the heterozygous genotype, the OR did not increase for the homozygous, and hence the association lacked a gene-dosage trend.

In Table 4

**Table 4:** Associations between homozygous CC genotype of +1127 *INS-Pst*I and characteristics of prostate cancer at diagnosis

**Dependent variable**	**Number of cases**	**Percent with homozygous CC**	**Univariate *P*-value**	**Adjusted OR (95% CI)**	**Multivariable *P*-value**
Gleason score					
2–6	116	84	0.034	2.60 (1.15–5.88)^a^	0.022
⩾7	52	69			
Age at diagnosis					
<55	26	69			
55–64	72	79			
⩾65	80	84	0.121^b^	2.10 (1.01–4.33)^c^	0.046

a Odds ratios for the risk of having a low Gleason score <7 were obtained from logistic regression analysis, when the subjects with homozygous CC were compared with those with other genotypes (reference category). Odds ratio was adjusted for age at diagnosis (<55, 55–64, or ⩾65) and frequency of seeing a doctor for physical examination (less than once a year, once a year, or more than once a year).

b *P* for trend.

c Odds ratios were estimated from ordinal logistic regression analysis. The positive odds ratio (2.10) indicates that the subjects with homozygous CC were more likely to be in a higher category of age at diagnosis (i.e. diagnosed at a later age) than subjects with other genotypes. The odds ratio was adjusted for Gleason score (2–6 or ⩾7) and frequency of seeing a doctor for physical examination.

, the case–case analyses showed that prostate cancer patients with the CC genotype, as compared to those with other genotypes, were more likely to have a low Gleason score < 7 (OR=2.60) after controlling for variables that were significantly associated with Gleason score, namely age at diagnosis and frequency of seeing a physician for physical examination. The CC genotype was also associated with a late age of diagnosis (OR=2.10) after adjusting for Gleason score and frequency of physical examination.

## DISCUSSION

The role of insulin in the aetiology of prostate cancer is implicated by the observations from this and another study in China that increased risk of prostate cancer was associated with genetic variation in *INS*, but not its neighbouring genes, as well as elevated fasting insulin levels (Hsing *et al*, 2001). Epidemiological studies have also found a positive correlation between insulin levels and risk of various cancers, such as colon, breast, and endometrial cancers (Maggino *et al*, 1993; Gamayunova *et al*, 1997; Troisi *et al*, 1997; Del Giudice *et al*, 1998; Schoen *et al*, 1999; Josefson, 2000; Kaaks *et al*, 2000; Yang *et al*, 2001; Goodwin *et al*, 2002). Biological mechanisms that support the tumorigenic effects of insulin include the following: (a) Insulin regulates and stimulates cell growth through binding to its receptor (Van Obberghen and Gammeltoft, 1986; Denton and Tavare, 1995; Moule and Denton, 1997). However, mitogenicity appears to occur at supraphysiologic levels of insulin. (b) It inhibits apoptosis in different cellular models (Park *et al*, 2000; Qian *et al*, 2001). (c) Insulin, IGF-I and IGF-II share about 50% structural homology, and there is 60% homology between insulin receptor and IGF-1R. Insulin and IGFs can crossbind to each other's receptor or to hybrid insulin and IGF-I receptors, although the affinity is weak (Soos *et al*, 1990; Efstratiadis, 1998). In some *in vitro* studies, the growth-promoting effects of insulin are mediated primarily by its low-affinity interaction with IGF-1R (Straus, 1984). (d) Insulin is the primary regulator of IGFBP-1. It inhibits transcription of IGFBP-1, and this may increase unbound, circulating IGFs (Powell *et al*, 1991). (e) Insulin decreases the synthesis of (SHBP) and may thereby increase the bioavailability of free steroids (e.g. testosterone) for hormone-dependent tissues like the prostate (Strain *et al*, 1994; Pasquali *et al*, 1995).

The +1127 *INS-Pst*I marker is located in the 3′ untranslated region (UTR) of *INS*, and the UTR regions of the preproinsulin mRNA have recently been demonstrated to play crucial roles in regulating insulin production. The 3′-UTR of *INS* suppresses translation and also stabilizes the mRNA. It acts cooperatively with the 5′-UTR and markedly increases glucose-induced proinsulin biosynthesis (Wicksteed *et al*, 2001). Therefore, the polymorphism at +1127 *INS-Pst*I, although located in an untranslated region, may have a functional effect on the expression of *INS*.

The +1127 *INS-Pst*I polymorphism may also be in intragenic linkage disequilibrium with a causal mutation in *INS*. In Caucasian populations in the US and Europe, the +1127 *INS-Pst*I polymorphism and nine other noncoding markers within the *INS* region are in tight linkage disequilibrium with the VNTR locus, which is located only 365 bp from the start of transcription for insulin (Cox *et al*, 1988; Julier *et al*, 1991; Lucassen *et al*, 1993). The class I allele of the VNTR is related to overexpression of insulin mRNA and increased insulin levels in some studies (Lucassen *et al*, 1995; Bennett and Todd, 1996; Le Stunff *et al*, 2000). The C allele of +1127 *INS-Pst*I, which was associated with increased risk of prostate cancer in this study, is linked with the class I allele of the VNTR. The extent of linkage disequilibrium across the insulin gene and VNTR, however, has never been studied in the Blacks or Hispanics. Hence it is not known if +1127 *INS-Pst*I is also a surrogate for the VNTR in the non-Whites in this study. Nevertheless, the significant linkage disequilibrium in the Caucasians suggests a strong evolutionary selection (Lucassen *et al*, 1993), and it is likely that strong linkage disequilibrium also exists in other populations as has been shown in a Chinese population (Cox *et al*, 1988).

Finally, there remains the possibility that *INS* is not the risk-associated gene, and that +1127 *INS-Pst*I is in linkage disequilibrium with another marker in a disease-causing gene. It is unlikely, since *INS* showed the strongest association with risk of prostate cancer, as the strength of association dropped off with the two neighbouring genes. It is, however, not surprising to see residual association between prostate cancer and the CT genotype of –4217 *TH-Pst*I (OR=2.27), since the +1127 *INS-Pst*I and –4217 *TH-Pst*I markers were in linkage disequilibrium.

If the polymorphism at +1127 *INS-Pst*I increases prostate cancer risk via altered insulin levels, this may explain its lack of association with prostate cancer among the diabetics. The insulin levels of the diabetics can be manipulated by medical intervention, rendering the genotype irrelevant for cancer risk. Other treatment for diabetes, such as weight reduction, could potentially modify the genetic susceptibility to prostate cancer. Since the +1127 *INS-Pst*I marker and its tightly linked VNTR are associated with the risk of diabetes and different alleles are involved in type I *vs*. type II diabetes (Julier *et al*, 1991; Lucassen *et al*, 1993; Bennett and Todd, 1996; Ong *et al*, 1999; Le Stunff *et al*, 2000), the allele frequencies of +1127 *INS-Pst*I could potentially be affected by the cause and type of diabetes in both the diabetic cases and controls. The association between prostate cancer and the +1127 *INS-Pst*I marker could be muddled in the diabetics – a heterogeneous group with diverse aetiology, type, and treatment of diabetes as well as endogenous and exogenous insulin. The nondiabetics were simply a group without the complications from a complex disease.

Unlike the Black and Caucasian years, a negative association between the CC genotype and prostate cancer was observed among the Hispanics. This could be because of small sample size or a population stratification bias. We examined for Hardy–Weinberg equilibrium of the genotype frequencies of +1127 *INS-Pst*I by the likelihood ratio test among the controls (Lange, 1997). Hardy–Weinberg equilibrium was found in both the Caucasians and Blacks, but not in the Hispanic population, in which the frequency of the homozygous TT was higher than expected (*P*<0.0001). Population admixture, one of the factors that could alter allele frequencies in the Hispanics, may cause biased results in association studies (Ewens and Spielman, 1995).

It is interesting that although the polymorphism at +1127 *INS-Pst*I increased the risk for prostate cancer, it was associated with favourable clinical characteristics of the tumour. After adjusting for frequency of physical examination by a clinician, a proxy for access to health care, the CC genotype was associated with late age at diagnosis and low Gleason score. The results suggest that genetic effects take time to accumulate and manifest while affecting the prostate tissue slowly. Alternatively, the age-dependent penetrance of *INS* may be elevated through interaction with some unknown ageing factors.

Our data suggest that the insulin gene plays a role in the aetiology of prostate cancer. Given the high prevalence of the homozygous CC genotype (>60% in this study population), its population attributable risk can be high. Moreover, given its association with late age of onset of low-grade prostate tumors, the polymorphism at +1127 *INS-Pst*I may contribute to the unique features of prostate cancer that are not seen in other cancers, namely the high prevalence of latent and indolent cancers and the dramatic increase in incidence with age.

Finally, although the polymorphisms of *INS* have been studied for their roles in diabetes and obesity (Julier *et al*, 1991; Lucassen *et al*, 1993; Bennett and Todd, 1996; Ong *et al*, 1999), this is the first published report on the association between the *INS* gene and risk of cancer. As a result of our small sample size, our results need to be replicated by population-based studies with a sample size sufficient to confirm various subgroup associations. Another limitation of this study is that fasting blood samples and hence insulin levels were not available. We were not able to examine the hypothesis that the disease association of the polymorphism at +1127 *INS-Pst*I is mediated through altered insulin levels. The available data in the literature have shown that (a) high insulin or C-peptide levels (an indicator for the pancreatic secretion of insulin) are associated with increased risk of cancer (e.g. prostate, colon, breast, and endometrial cancer) (Maggino *et al*, 1993; Gamayunova *et al*, 1997; Troisi *et al*, 1997; Del Giudice *et al*, 1998; Schoen *et al*, 1999; Josefson, 2000; Kaaks *et al*, 2000; Hsing *et al*, 2001; Yang *et al*, 2001; Goodwin *et al*, 2002), (b) there is a relationship between the VNTR allelic variation and *INS* expression and/or production (Lucassen *et al*, 1995; Bennett and Todd, 1996; Le Stunff *et al*, 2000), and (c) there appears to be an association between a SNP tightly linked with the VNTR and the risk of prostate cancer. Future studies on cancer should examine comprehensively the inter-relationships among fasting insulin levels, insulin sensitivity, polymorphisms of the *INS* gene (either the VNTR or one of the surrogate SNPs), and risk of cancer.
